# Estimating the local false discovery rate via a bootstrap solution to the reference class problem

**DOI:** 10.1371/journal.pone.0206902

**Published:** 2018-11-26

**Authors:** Farnoosh Abbas-Aghababazadeh, Mayer Alvo, David R. Bickel

**Affiliations:** 1 Department of Mathematics and Statistics, University of Ottawa, Ottawa, Ontario, Canada; 2 Ottawa Institute of Systems Biology Biochemistry, Microbiology, and Immunology Department, University of Ottawa, Ottawa, Ontario, Canada; 3 Department of Biostatistics and Bioinformatics, Moffitt Cancer Center, Tampa, FL, United States of America; UCLA, UNITED STATES

## Abstract

Methods of estimating the local false discovery rate (LFDR) have been applied to different types of datasets such as high-throughput biological data, diffusion tensor imaging (DTI), and genome-wide association (GWA) studies. We present a model for LFDR estimation that incorporates a covariate into each test. Incorporating the covariates may improve the performance of testing procedures, because it contains additional information based on the biological context of the corresponding test. This method provides different estimates depending on a tuning parameter. We estimate the optimal value of that parameter by choosing the one that minimizes the estimated LFDR resulting from the bias and variance in a bootstrap approach. This estimation method is called an *adaptive reference class* (ARC) method. In this study, we consider the performance of ARC method under certain assumptions on the prior probability of each hypothesis test as a function of the covariate. We prove that, under these assumptions, the ARC method has a mean squared error asymptotically no greater than that of the other method where the entire set of hypotheses is used and assuming a large covariate effect. In addition, we conduct a simulation study to evaluate the performance of estimator associated with the ARC method for a finite number of hypotheses. Here, we apply the proposed method to coronary artery disease (CAD) data taken from a GWA study and diffusion tensor imaging (DTI) data.

## 1 Introduction

Methods of estimating the local false discovery rate (LFDR) [1], not suffering from the bias inherent in estimating other false discovery rates [2], have been applied to various datasets such as high-throughput biological data (e.g., gene expression, proteomics, and metabolomics), diffusion tensor imaging (DTI), and genome-wide association (GWA) study [3–5]. As an example, in a GWA study, the methods of estimating the LFDR are used in order to estimate the probability that a single nucleotide polymorphism (SNP) is associated with a disease [6–10]. In addition, in DTI brain scans, the LFDR estimates have been used to estimate the proportion of dyslexic-non-dyslexic differences [5, 11].

In many situations, the considered hypotheses are connected by a scientific context. However, ignorance of this scientific context in a data analysis can be misleading, because it may introduce bias into the LFDR estimates [11]. For example, each test in a GWA study corresponds to a specific genetic marker for which previous biological information may be available. Moreover, in a DTI study, each test corresponds to a voxel, where the voxel location can be incorporated as a scientific context.

### 1.1 Motivating example

**Fig 1:**
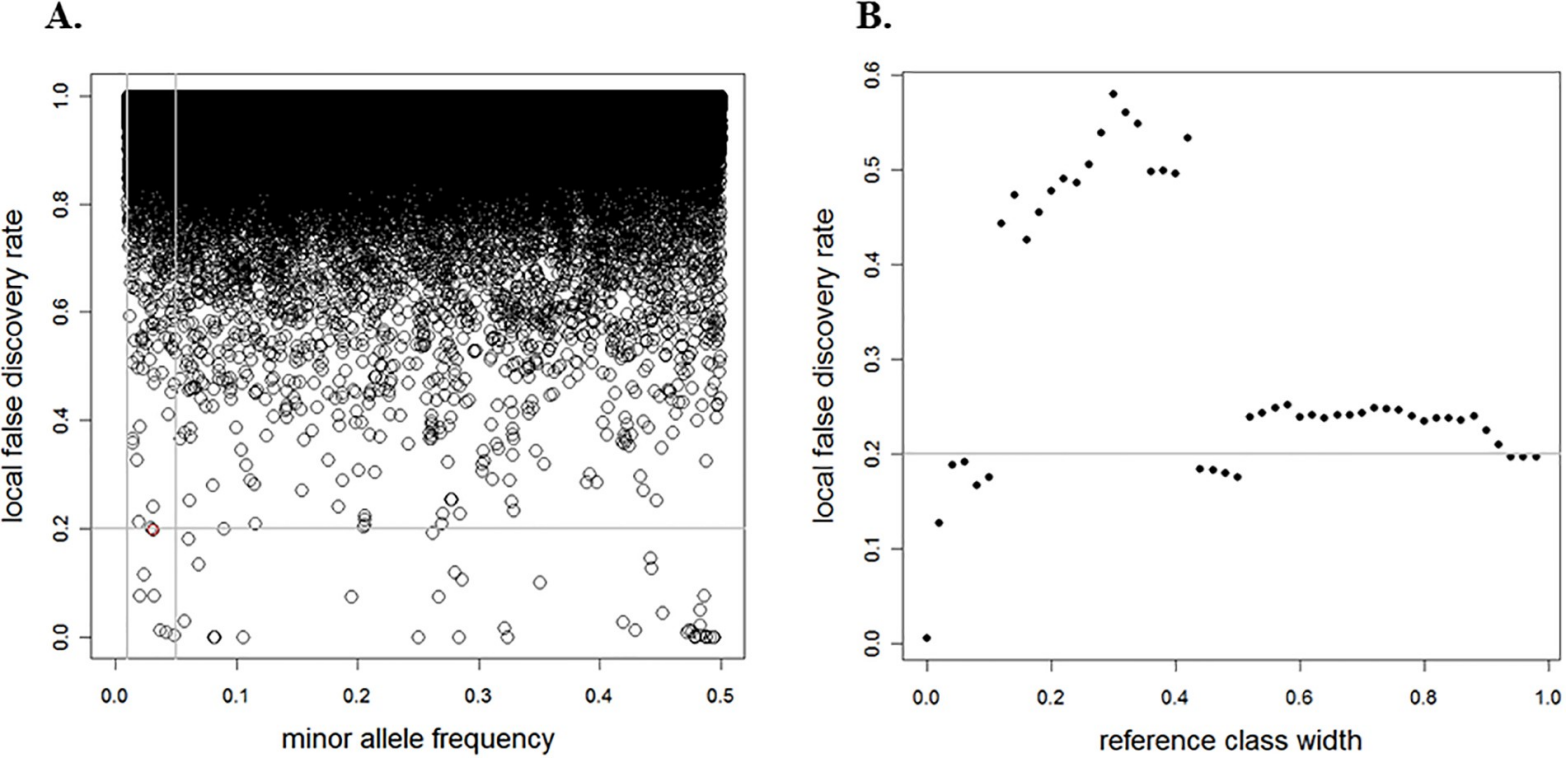
CAD data for LFDR estimates under the HB model versus the MAF *x*_*i*_ (A) and reference class width (B) for a fixed MAF *x*_*i*_ = 0.0306. The horizontal line represents the threshold of 0.2. The vertical lines in (A) indicate the symmetry around *x*_*i*_ = 0.0306 with Δ = 0.04.

We consider coronary artery disease (CAD) data [12], where *N* = 394, 839 SNPs passed the quality control (QC) filtering methods explained in Section 3.2.1. The aim of this study is to identify whether each SNP is associated with a disease. We have two components (*z*_*i*_, *x*_*i*_) associated with each hypothesis for *i* = 1, …, *N*, where *z*_*i*_ is an observed test statistic and *x*_*i*_ presents the minor allele frequency (MAF). For our data, all observed test statistics are used to identify the disease-associated SNPs. Fig 1(A) the *N* = 394, 839 SNPs used to estimate the LFDR (Section 2). A total of 44 disease-associated SNPs with LFDR estimates lower than 0.2 are identified.

A set of hypotheses or features used to determine the posterior probability of a null hypothesis is called a *reference class*, and the problem of finding such a set is an example of the reference class problem (e.g., Bickel [13]). For example, considering all SNPs when estimating the LFDR for a specific MAF, instead of considering the different subsets of SNPs, is called the *combined reference class* (CRC) method (Section 2). From Fig 1(A), consider the example of *x*_*i*_ = 0.0306 and *z*_*i*_ = −4.3971, with an estimated LFDR of 0.1973 that is close to the threshold of 0.2. For this MAF, we define a reference class of SNPs in such a way that the MAFs are within a symmetric window around *x*_*i*_ = 0.0306, with width 2Δ. Different window widths yield different reference classes. Again, each subset of SNPs is used to estimate the LFDR. Fig 1(B) illustrates the LFDR estimates versus the reference class width. This figure shows that changing the reference class width provides different LFDR estimates, raising the important question of how we can estimate the optimal reference class in order to estimate the LFDR. For the considered CAD data, the reference class problem consists of deciding which SNPs should be used to determine whether a SNP is associated with a disease.

The hypotheses can be divided into groups based on the characteristics of the problem. For example, the CAD data can be divided into two distinct groups according to MAFs, low-frequency SNPs (1% ≤ *x*_*i*_ ≤ 5%) and common SNPs (*x*_*i*_ > 5%). Thus, we need to determine which reference class should be used to determine the posterior probability that the SNP is not associated with the disease occurring at the MAF *x*_*i*_ = 0.0306. Should we use the entire set of SNPs, or the low-frequency SNPs [14]? In addition, SNPs can be divided into different classes, such as non-synonymous SNPs, genic SNPs, SNPs in highly conserved regions, SNPs in linkage disequilibrium with many (or few) other SNPs, or categorized SNPs based on their MAFs [12].

### 1.2 Previous research on the reference class problem

Many methods have been proposed for incorporating covariates into statistical techniques for testing multiple hypotheses. Bickel [15] considered the effect of selecting test statistics in estimators of the weighted and unweighted FDR, and found that smaller reference classes of null hypotheses yield lower estimated expected losses than larger reference classes do. Several researchers have applied the idea of incorporating a group structure and weights to improve the statistical power of tests. This group structure can be used when testing multiple hypotheses by assigning weights for the hypotheses or the *p*-values in each group. Benjamini and Hochberg [16] used a *p*-value weighting method to evaluate different procedures. Genovese et al. [17] demonstrated that a *p*-value weighting procedure can be employed to control the FWER and FDR while increasing the statistical power of the test. Subsequently, Wasserman and Roeder [18] introduced an optimal *p*-value weighting procedure to control the FWER. Sun et al. [19] proposed a stratified false discovery control approach for genetic studies, in which a large number of hypotheses include inherent stratification. In addition, Efron [11] argued that analyzing separate reference classes can be legitimate from a frequentist viewpoint, and Hu et al. [20] proposed a weighting scheme based on a simple Bayesian framework that employs the proportion of null hypotheses that are true within each group. Such an approach can control the FDR for *p*-values with certain dependence structures. The unknown proportion of true null hypotheses is estimated within each group. Moreover, Zablocki et al. [8] used a hierarchical Bayesian approach to incorporate a set of covariates, where the prior probability that the null hypothesis test is true and the alternative distribution of the test statistic are both modulated by covariates. In contrast to Zablocki et al. [8], instead of specifying the hyperprior distributions required for a hierarchical Bayesian approach, we follow Karimnezhad and Bickel [21] and use an empirical Bayes approach to estimate an optimal reference class to improve the LFDR estimate.

In this study, we assume the prior probability to be a function of covariates (Section 2.1). Then, we propose an adaptive reference class (ARC) method for estimating the LFDR, using a bootstrap approach to estimate the optimal reference class (Section 2.2). We compare the performance of the proposed ARC method and the CRC method using the mean squared error (MSE) as the performance criterion. We prove that, under certain assumptions, the ARC method has an MSE that is asymptotically no greater than that of the CRC method (see S1 File). In addition to the asymptotic results, we conduct a simulation study to investigate the finite dataset performances of the LFDR estimators for each method in Section 3.1. Both the asymptotic and simulation results show that, under certain assumptions, the ARC method performs well compared with the CRC method. We present an application of the ARC method on both CAD data and DTI data in Section 3.2 in order to demonstrate the practical importance of deciding between the ARC and CRC methods. Finally, we conclude the paper with a brief discussion in Section 4. The proof for the main theorem is included in S1 File.

## 2 Materials and methods

Suppose *N* null hypotheses *H*_01_,…, *H*_0*N*_ are considered simultaneously. For example in GWA study, let *H*_0*i*_ denote the null hypothesis that the *i*^th^ SNP is not associated with the disease. Under the genetic additive model [22], each SNP yields a *Wald* *χ*^2^ test statistic *W*_*i*_. Under the *i*^th^ null hypothesis, it holds that $W_{i}∼\chi_{1}^{2}$, while under the *i*^th^ alternative hypothesis, we have $W_{i}∼\chi_{1,\delta }^{2}$, where *δ* ∈ (0, ∞) is an unknown noncentrality parameter, following the models employed in [23] and [24]. Under the *i*^th^ null hypothesis, assume that *Z*_*i*_ ∼ *N*(0, 1), where *Z*_*i*_ represents the z-transform that converts the *Wald* *χ*^2^ statistic into a standard normal statistic. In addition, for DTI data, let *H*_0*i*_ denote the null hypothesis that there is no dyslexic-non-dyslexic difference for the *i*^th^ voxel. Under the *i*^th^ null hypothesis, assume that *Z*_*i*_ ∼ *N*(0, 1), where *Z*_*i*_ represents the z-transform which converts two-sample *t*-test satistic into a standard normal statistic.

The observed statistics ***z*** = (*z*_1_,…, *z*_*N*_)^*T*^ are considered realizations of ***Z*** = (*Z*_1_,…, *Z*_*N*_)^*T*^. Let *A*_*i*_ be an indicator variable for the event that the *i*^th^ alternative hypothesis *H*_*ai*_ is true. Assume that *A*_*i*_’s are independent and identically distributed (i.i.d.) Bernoulli(1 − *π*_0_) variables, where *π*_0_ is the prior probability that the *i*^th^ null hypothesis is true. Let *f*_0_(*z*_*i*_) and *f*_1_(*z*_*i*_) be the null and alternative density, respectively.

The posterior probability that the *i*^th^ null hypothesis is true, given *Z*_*i*_ = *z*_*i*_ is the LFDR [1], and is denoted as Ψ(*z*_*i*_), where

$$\Psi (z_{i})=\text{P}(A_{i}=0|Z_{i}=z_{i})=\frac{\pi_{0}f_{0}\left(z_{i}\right)}{f(z_{i};\pi_{0})},$$ (1)

where *f*(*z*_*i*_; *π*_0_) denotes the mixture density of *Z*_*i*_ given by

$$f(z_{i};\pi_{0})=\pi_{0}f_{0}\left(z_{i}\right)+(1-\pi_{0})f_{1}\left(z_{i}\right).$$ (2)

If the null hypothesis is true, then the null density *f*_0_(*z*_*i*_) of the statistic *Z*_*i*_ is the standard normal, and is called the *theoretical null hypothesis* [1]. The model defined in Eq (2) with the following method of estimation is called the *histogram- based* (HB) model [25]. By assuming the theoretical null hypothesis and applying the Poisson regression, the mixture density *f*(*z*_*i*_;*π*_0_) is estimated by fitting a high-degree polynomial to the histogram counts, denoted by ${\hat{f}}_{i}\left(z\right)$, where the estimate of the proportion of true null hypothesis is denoted by ${\hat{\pi }}_{0}\left(z\right)$. The LFDR estimate ${\hat{\Psi }}_{i}\left(z\right)$ is computed by substituting ${\hat{\pi }}_{0}\left(z\right)$ and ${\hat{f}}_{i}\left(z\right)$ into Eq (1). This model and estimation method are an example of the CRC method.

### 2.1 Proposed model

The described model in Eq (1) extends to the situation that incorporates a covariate related to the scientific context of each hypothesis test. For the CAD dataset, the covariate represents the MAF in the CAD data, while for the DTI data, the location is incorporated as a covariate. Let ***X*** = (*X*_1_,…, *X*_*N*_)^*T*^ be i.i.d. random variables. Any test statistics are transformed to the standard normal statistic *Z*_*i*_, for *i* = 1,…, *N*. The observed statistics vector ***z*** = (*z*_1_,…, *z*_*N*_)^*T*^ is considered a realization of ***Z*** = (*Z*_1_,…, *Z*_*N*_)^*T*^. Let *A*_*i*_ be the event that the *i*^th^ alternative hypothesis *H*_*ai*_ is true. Assume that *A*_*i*_|*X*_*i*_ = *x*_*i*_ ∼ Bernoulli(1 − *π*_0_(*x*_*i*_)), where *π*_0_(*x*_*i*_), the prior probability that the *i*^th^ null hypothesis is true, is an unknown function of the given covariate *X*_*i*_ = *x*_*i*_. We denote the posterior probability that the *i*^th^ null hypothesis is true, given *Z*_*i*_ = *z*_*i*_ and *X*_*i*_ = *x*_*i*_ by

$$\Psi (z_{i};x_{i})=\text{P}(A_{i}=0|Z_{i}=z_{i},X_{i}=x_{i})=\frac{\pi_{0}\left(x_{i}\right)f_{0}\left(z_{i}\right)}{f(z_{i};\pi_{0}\left(x_{i}\right))},$$ (3)

where the mixture density of *Z*_*i*_ conditional on the covariate *X*_*i*_ = *x*_*i*_ is given by

$$f(z_{i};x_{i})=\pi_{0}\left(x_{i}\right)f_{0}\left(z_{i}\right)+(1-\pi_{0}\left(x_{i}\right))f_{1}\left(z_{i};x_{i}\right),$$ (4)

where *f*_0_(*z*_*i*_) denotes the null density of *Z*_*i*_ and *f*_1_(*z*_*i*_;*x*_*i*_) is the alternative density of *Z*_*i*_. The mixture density in Eq (2) is a special form of Eq (4), where the effect of the covariates is ignored. The quantities *π*_0_(*x*_*i*_) and *f*_1_(*z*_*i*_;*x*_*i*_) are unknown. The ARC method is applied to estimate the LFDR in Eq (3). Under the CRC method, the effects of the covariates defined in Eq (1) are ignored, while under the proposed ARC method, some assumptions are considered locally in order to estimate the LFDR Ψ(*z*_*i*_; *x*_*i*_), defined in Eq (3).

### 2.2 Adaptive reference class (ARC) method

Under the ARC method, certain assumptions only hold locally within a symmetric window for each covariate. Let a symmetric window of width 2Δ be centered at given covariate *X*_*i*_ = *x*_*i*_. Such a symmetric window is denoted by $z_{i}^{\Delta }$, where

$$z_{i}^{\Delta }=\{z_{j}:\;|x_{j}-x_{i}\left|\leq \Delta ,\;j=1,\ldots ,N\right\}.$$ (5)

Let Δ_0_ denote the smallest considered value of the tuning parameter Δ. The reference class $z_{i}^{\Delta }$ contains components *z*_*j*_ such that their covariates are within a distance Δ of *x*_*i*_. Denoting the expected dimension of the reference class $z_{i}^{\Delta }$ by ${\text{d}}_{i}^{\Delta }$, we have

$${\text{d}}_{i}^{\Delta }=N\text{P}(|X_{j}-x_{i}|\leq \Delta ,\;j=1,\ldots ,N).$$ (6)

Here, ${\text{d}}_{i}^{\Delta }$ increases with number of null hypothesis tests *N*, provided that the probability is positive. For each reference class $z_{i}^{\Delta }$, we may apply any LFDR estimation approach such as the HB model in Section 2. In contrast to the CRC method, instead of using the entire collection of observed statistics ***z***, only the reference class $z_{i}^{\Delta }$ is used to obtain the LFDR estimate ${\hat{\Psi }}_{i}\left(z_{i}^{\Delta }\right)$. The choice of tuning parameter Δ influences the LFDR estimate. Here, we choose the one that results in the lowest error when estimating the LFDR, which is called the *optimal* tuning parameter.

### 2.3 Optimal tuning parameter

The optimal tuning parameter Δ specifies the symmetric window width of a given reference class, and is determined by minimizing the errors resulting from the bias and the variance. In the following, we introduce several notational conventions.

Let the mean and variance of the estimator ${\hat{\Psi }}_{i}\left(z_{i}^{\Delta }\right)$ be defined as

$$\begin{array}{cc} \mu_{\Delta }\left(x_{i}\right)= & \text{E}({\hat{\Psi }}_{i}\left(z_{i}^{\Delta }\right)|X_{i}=x_{i}),\\ \sigma_{\Delta }^{2}\left(x_{i}\right)= & \text{E}[({\hat{\Psi }}_{i}\left(z_{i}^{\Delta }\right)-\mu_{\Delta }\left(x_{i}\right){)}^{2}|X_{i}=x_{i}], \end{array}$$ (7)

respectively. When *X*_*i*_ = *x*_*i*_, the prediction bias for the estimator ${\hat{\Psi }}_{i}\left(z_{i}^{\Delta }\right)$ is denoted by $B_{\Delta }\left(x_{i}\right)$, with

$$B_{\Delta }\left(x_{i}\right)=\text{E}[({\hat{\Psi }}_{i}\left(z_{i}^{\Delta }\right)-\Psi \left(z_{i};x_{i}\right))|X_{i}=x_{i}].$$ (8)

Determining the optimal choice of Δ depends on the choice of the loss function used to measure the errors in the estimation of the LFDR. A good estimator is accurate in the sense that its estimates are as close to the true values as possible. Accuracy measures typically take into account the difference between the estimated value and the true value. Using the MSE as a an accuracy measure is a commonly used way to indicate how close the estimator is to the true value by incorporating both the bias and the variance [26]. Hence, using the MSE provides our criterion for defining the optimal tuning parameter Δ. The MSE for the estimator ${\hat{\Psi }}_{i}\left(z_{i}^{\Delta }\right)$ conditional on *X*_*i*_ = *x*_*i*_ is defined as

$$\text{MSE}({\hat{\Psi }}_{i}\left(z_{i}^{\Delta }\right)|X_{i}=x_{i})=\text{E}[({\hat{\Psi }}_{i}\left(z_{i}^{\Delta }\right)-\Psi \left(z_{i};x_{i}\right){)}^{2}|X_{i}=x_{i}].$$ (9)

It can be shown that the portion of MSE that depends on Δ is given by

$$\text{err}({\hat{\Psi }}_{i}\left(z_{i}^{\Delta }\right)|X_{i}=x_{i})=\sigma_{\Delta }^{2}\left(x_{i}\right)+B_{\Delta }^{2}\left(x_{i}\right).$$ (10)

Here, we employ the errors resulting from the bias and the variance in Eq (10) to determine the optimal Δ. Denoting the optimal Δ by $\Delta_{0i}^{⋆}$, we have

$$\Delta_{0i}^{⋆}=\text{arg}\underset{\Delta \geq \Delta_{0}}{\text{inf}}\text{err}({\hat{\Psi }}_{i}\left(z_{i}^{\Delta }\right)|X_{i}=x_{i}).$$ (11)

To estimate $\Delta_{0i}^{⋆}$, it is necessary to estimate the variance and the prediction bias of the LFDR estimator, which we do using the bootstrap approach.

### 2.4 Bootstrap estimation of the optimal tuning parameter

We re-sample *N* pairs from {(*z*_1_, *x*_1_),…, (*z*_*N*_, *x*_*N*_)} until *B* bootstrap samples are obtained that contain the specific pair (*z*_*i*_, *x*_*i*_), where *z*_*i*_ ∈ ***z*** and *x*_*i*_ ∈ ***x***. These samples are denoted by $\left(z_{1}^{⋆},x_{1}^{⋆}\right),\ldots ,\left(z_{B}^{⋆},x_{B}^{⋆}\right)$. The *b*^th^ bootstrap sample $\left(z_{b}^{⋆},x_{b}^{⋆}\right)$ contains pairs $\left(z_{bj}^{⋆},x_{bj}^{⋆}\right)$, for *j* = 1,…, *N* and *b* = 1,…, *B*. From Eq (5), the *b*^th^ bootstrap reference class is defined as

$$z_{i,b}^{\Delta }=\{z_{bj}^{⋆}:\;|x_{bj}^{⋆}-x_{i}\left|\leq \Delta ,\;j=1,\ldots ,N\right\}.$$ (12)

The estimate of Ψ(*z*_*i*_; *x*_*i*_) based on the *b*^th^ bootstrap reference class is denoted by ${\hat{\Psi }}_{i}\left(z_{i,b}^{\Delta }\right)$. The random variables ${\hat{\Psi }}_{i}\left(z_{i,1}^{\Delta }\right),\ldots .,{\hat{\Psi }}_{i}\left(z_{i,B}^{\Delta }\right)$ provide the estimators $\hat{\mu }\left(\Delta ,B\right)$ and ${\hat{\sigma }}^{2}\left(\Delta ,B\right)$, which we use to estimate *μ*_Δ_(*x*_*i*_) and $\sigma_{\Delta }^{2}\left(x_{i}\right)$, respectively, where

$$\hat{\mu }\left(\Delta ,B\right)=\frac{1}{B}\sum_{b=1}^{B}{\hat{\Psi }}_{i}\left(z_{i,b}^{\Delta }\right)\;\;\text{and}\;\;{\hat{\sigma }}^{2}\left(\Delta ,B\right)=\frac{1}{B-1}\sum_{b=1}^{B}({\hat{\Psi }}_{i}\left(z_{i,b}^{\Delta }\right)-\hat{\mu }\left(\Delta ,B\right){)}^{2}.$$ (13)

In order to estimate the prediction bias in Eq (10), we need to estimate *π*_0_(*x*_*i*_). We propose using a reference class $z_{i,b}^{\Delta_{0}}$, which contains the observed statistics *z*_*j*_s the covariates of which are within a distance Δ_0_ of *x*_*i*_. Thus, the estimator $\hat{\mu }\left(\Delta_{0},B\right)$ from Eq (13) can be used to estimate *π*_0_(*x*_*i*_). Denoting the bootstrap estimator of the prediction bias by $\hat{B}\left(\Delta ,\Delta_{0},B\right)$, we have that

$$\hat{B}\left(\Delta ,\Delta_{0},B\right)=\hat{\mu }\left(\Delta ,B\right)-\hat{\mu }\left(\Delta_{0},B\right).$$ (14)

The estimator of $\text{err}({\hat{\Psi }}_{i}\left(z_{i}^{\Delta }\right)|X_{i}=x_{i})$ in Eq (10) is denoted by $\hat{\text{err}}\left(\Delta ,\Delta_{0},B\right)$, and is computed by simply summing the bootstrap variance in Eq (13) and the squared bootstrap prediction bias in Eq (14). Let the optimal $\Delta_{0i}^{⋆}$ be denoted as ${\hat{\Delta }}_{0i}^{⋆}$, which is given by

$${\hat{\Delta }}_{0i}^{⋆}=\text{arg}\underset{\Delta \geq \Delta_{0}}{\text{inf}}\hat{\text{err}}\left(\Delta ,\Delta_{0},B\right).$$ (15)

After estimating the optimal tuning parameter ${\hat{\Delta }}_{0i}^{⋆}$ (see Algorithm 1), the optimal reference class $z_{i}^{{\hat{\Delta }}_{0i}^{⋆}}$ is estimated from Eq (5). The class contains *z*_*j*_s, the covariates of which are within a distance ${\hat{\Delta }}_{0i}^{⋆}$ of *x*_*i*_. Then, the optimal reference class $z_{i}^{{\hat{\Delta }}_{0i}^{⋆}}$ is used to estimate the LFDR in Eq (3). This LFDR estimate is denoted as ${\hat{\Psi }}_{i}(z_{i}^{{\hat{\Delta }}_{0i}^{⋆}})$. The estimation methods detailed above yield two estimators. The estimator ${\hat{\Psi }}_{i}\left(z\right)$ is related to the CRC method and ${\hat{\Psi }}_{i}\left(z_{i}^{{\hat{\Delta }}_{0i}^{⋆}}\right)$ is computed using the ARC method. We compare the performance of two estimators using the MSE.

**Algorithm 1** Pseudo-code of the estimation of the optimal tuning parameter for one simulated dataset.

**Input**: Test statistics and covariates (*z*_1_, *x*_1_),…, (*z*_*N*_, *x*_*N*_); number of bootstrap samples *B*; smallest value of the tuning parameterΔ_0_; tuning parameter Δ ≥ Δ_0_

For *i* = 1,…*N*

- Build *b*^*th*^ bootstrap samples $\left(z_{b}^{⋆},x_{b}^{⋆}\right)=\left\{\left(z_{bj}^{⋆},x_{bj}^{⋆}\right),\ldots ,\left(z_{bj}^{⋆},x_{bj}^{⋆}\right)\right\}$, *b* = 1,…, *B* and *j* = 1,…, *N* including pair (*z*_*i*_, *x*_*i*_) For *b* = 1,…, *B*
  - Determine bootstrap reference class $z_{i,b}^{\Delta_{0}}$ and $z_{i,b}^{\Delta }$ in Eq (12)
  - Estimate LFDR ${\hat{\Psi }}_{i}\left(z_{i,b}^{\Delta }\right)$ and ${\hat{\Psi }}_{i}\left(z_{i,b}^{\Delta_{0}}\right)$ using HB method
- Compute $\hat{\mu }\left(\Delta ,B\right)$, ${\hat{\sigma }}^{2}\left(\Delta ,B\right)$, $\hat{\mu }\left(\Delta_{0},B\right)$, $\hat{B}\left(\Delta ,\Delta_{0},B\right)$ and $\hat{\text{err}}\left(\Delta ,\Delta_{0},B\right)$ in Eqs (10), (13) and (14)
- Minimize $\hat{\text{err}}\left(\Delta ,\Delta_{0},B\right)$ over Δ ≥ Δ_0_

**Output**: Estimate optimal tuning parameter ${\hat{\Delta }}_{0i}^{⋆}$.

Let *π*_0_(*x*_*i*_) denote the true prior probability that the *i*^th^ null hypothesis is true. In GWA study, the null hypothesis means no disease association, and in the DTI study, it means no differences between dyslexic and non-dyslexic children. For a given *x*_0_, we suppose that the unknown prior probability *π*_0_(*X*_*i*_) is a step function of the covariate *X*_*i*_, given by

$$\pi_{0}\left(X_{i}\right)=\{\begin{array}{cc} \pi_{01} & \;\;\text{if}\;X_{i}\leq x_{0},\\ \pi_{02} & \;\;\text{if}\;X_{i}>x_{0}, \end{array}$$ (16)

where the prior probabilities *π*_01_ and *π*_02_ are both unknown, and *π*_01_ ≤ *π*_02_. This function splits the *N* tests into two distinct groups, such that in each group, the test statistics are i.i.d. Moreover, the simplified function in Eq (16) will have a biologically meaningful interpretation. As an example, in CAD data, the N SNPs can be divided into two distinct groups; disease-associated and not disease-associated. The two groups may have different choices of prior probabilities. In addition, under the assigned values of *x*_0_ and Δ_0_, the observed vector of covariates ***x*** may be partitioned into three regions

$$\begin{array}{cc} R_{1}\left(x_{0},\Delta_{0}\right)= & \{x_{j}:\;x_{j}\leq x_{0}-\Delta_{0}\},\\ R_{2}\left(x_{0},\Delta_{0}\right)= & \{x_{j}:\;x_{0}-\Delta_{0}<x_{j}<x_{0}+\Delta_{0}\},\\ R_{3}\left(x_{0},\Delta_{0}\right)= & \{x_{j}:\;x_{j}\geq x_{0}+\Delta_{0}\}, \end{array}$$ (17)

for *j* = 1,.., *N*. Therefore, the following theorem ensures us that, under the assumptions explained above for *π*_0_(*X*_*i*_) Eq (16) and the region of covariates Eq (17), the proposed ARC method has an MSE that is asymptotically no greater than that of the CRC method. The proof of the theorem is given as a series of lemmas (see S1 File).

**Theorem 1** *Let* ${\hat{\Psi }}_{i}\left(z\right)$ *be a weakly consistent estimator of* Ψ(*z*_*i*_) *when N becomes large*. *If* $x_{i}\in R_{1}\left(x_{0},\Delta_{0}\right)$, *then*

$$\underset{N\to \infty }{\text{lim}}\underset{B\to \infty }{\text{lim}}[\text{MSE}({\hat{\Psi }}_{i}\left(z_{i}^{{\hat{\Delta }}_{0i}^{⋆}}\right)|R_{1}\left(x_{0},\Delta_{0}\right))-\text{MSE}({\hat{\Psi }}_{i}\left(z\right)|R_{1}\left(x_{0},\Delta_{0}\right))]\leq 0.$$

*where B is the number of bootstrap samples*.

**Remark 1** *The step function of the covariates in* Eq (16) *and the partitioning of the covariates in* Eq (17) *affect the weak consistency of* $\hat{\mu }\left(\Delta_{0},B\right)$ Eq (13) *as an estimator of π*_0_(*X*_*i*_) *in* Eq (16) (*see* S1 File, *Lemma 2*). *Then*, *for* $x_{i}\in R_{2}\left(x_{0},\Delta_{0}\right)$, *the bootstrap mean* $\hat{\mu }\left(\Delta_{0},B\right)$ *is not a weakly consistent estimator of π*_0_(*X*_*i*_).

**Remark 2** *For* $x_{i}\in R_{3}\left(x_{0},\Delta_{0}\right)$, *similar results to Theorem 1 can be derived*.

## 3 Results

### 3.1 Simulation study

The aim of the simulation analysis presented here is to compare the finite dataset performances of the CRC and ARC methods when estimating the LFDR in Eq (3). In this section, each test statistic is assigned a prior probability that is a function of the covariate.

We assume that the proportion of disease-associated tends to be very small. Then, we present several simulation studies, each with a different value of *x*_0_ ∈ [0.05, 0.40]. The datasets are simulated as follows. In each simulation, we randomly generate 1000 datasets, each corresponding to an artificial case-control study. For each dataset, we simultaneously generate both the auxiliary information and the observed Wald *χ*^2^ test statistics, denoted by *x*_*i*_ and *w*_*i*_, respectively. Each observed covariate *x*_*i*_ is generated randomly from the uniform distribution between 0 and 1.

In each simulation, the true prior probability *π*_0_(*x*_*i*_) is determined according the given value of *x*_0_ as a function of the observed covariate in Eq (16). From (16), let ${\bar{\pi }}_{0}=\text{E}\left(\pi_{0}\left(X_{i}\right)\right)$ for *i* = 1,…, *N*, where ${\bar{\pi }}_{0}\in \left[0.60,0.95\right]$. To generate the observed statistics, we generate each *A*_*i*_ ∼ Bernoulli(1 − *π*_0_(*x*_*i*_)) independently. To generate the observed *χ*^2^ test statistics, if *A*_*i*_ = 1, the observed statistics are sampled from $\chi_{1,\delta }^{2}$ with a noncentrality parameter *δ*. For each given value of *x*_0_, a different value of *δ* ∈ [1.5, 7] is assigned. The Wald *χ*^2^ test statistics when *A*_*i*_ = 0 are sampled from $\chi_{1}^{2}$. The *Wald* *χ*^2^ test statistics are then transformed into *z*-values.

**Fig 2:**
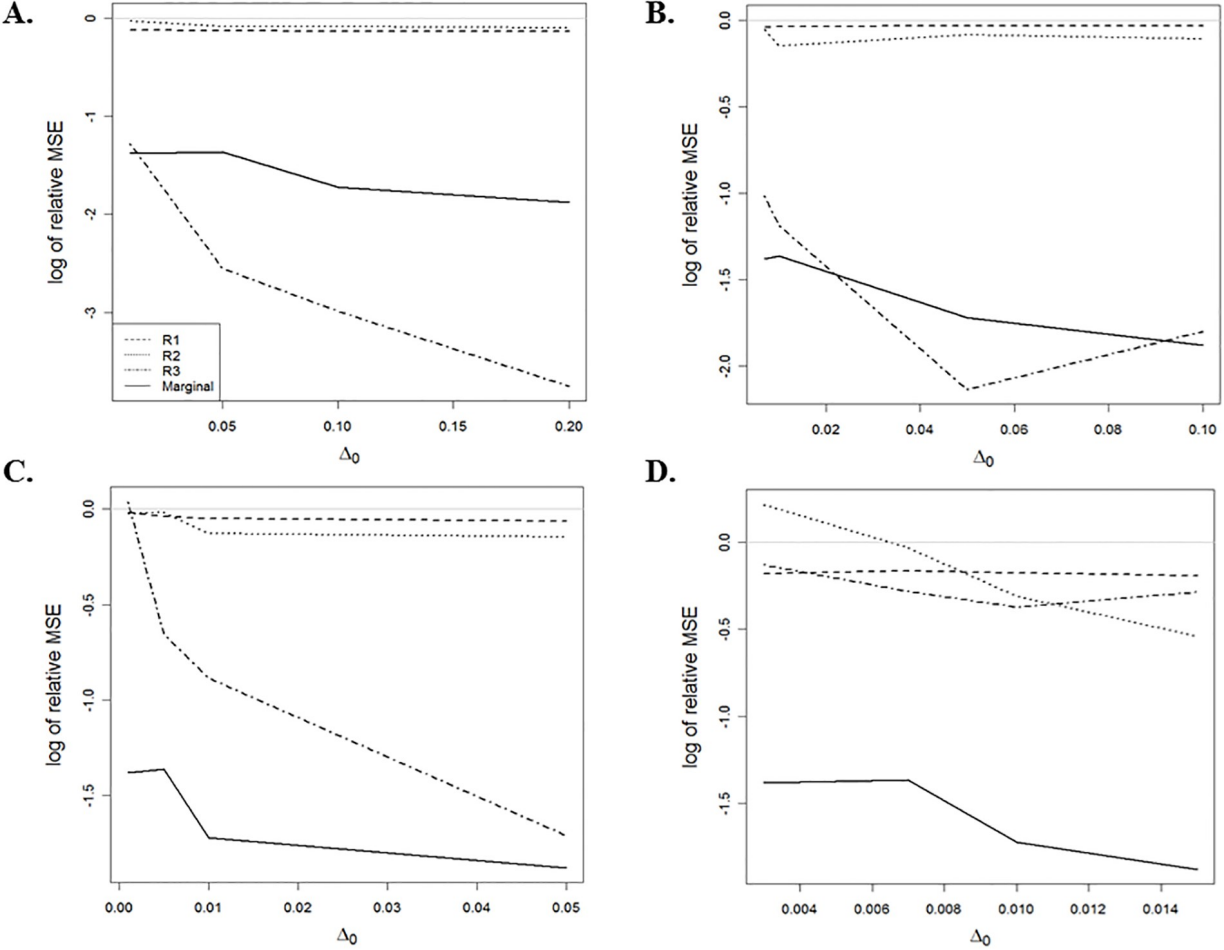
The log_2_ value of the relative MSE conditional on regions and marginal versus different values of Δ_0_; for (A) ${\bar{\pi }}_{0}=0.60$, (B) ${\bar{\pi }}_{0}=0.80$, (C) ${\bar{\pi }}_{0}=0.90$ and (D) ${\bar{\pi }}_{0}=0.95$, where ${\bar{\pi }}_{0}=\text{E}\left(\pi_{0}\left(X_{i}\right)\right)$ for *i* = 1, …, *N*.

Each dataset has *N* pairs (*z*_*i*_, *x*_*i*_). The total number of pairs *N* is equal to 300, 000. In each simulation, a pair (*z*_*i*_, *x*_*i*_) is selected randomly from each dataset to estimate Ψ(*z*_*i*_; *x*_*i*_). For a given covariate *x*_*i*_, the estimators of the LFDR are computed using the two methods. Under the ARC method, Δ_0_ has to be specified in advance in order to determine ${\hat{\Delta }}_{0i}^{⋆}$. We consider the range Δ_0_ ∈ (0, *x*_0_), and set *B* = 1000. Thus, under the HB model described in Section 1.1, we compute the estimators ${\hat{\Psi }}_{i}\left(z\right)$ and ${\hat{\Psi }}_{i}\left(z_{i}^{{\hat{\Delta }}_{0i}^{⋆}}\right)$. The conditional MSE approximations used to measure the performances of the estimators are given by

$$\hat{\text{MSE}}({\hat{\Psi }}_{i}|R_{r}\left(x_{0},\Delta_{0}\right))=\frac{1}{\#\{x_{i}\in R_{r}\left(x_{0},\Delta_{0}\right)\}}\sum_{x_{i}\in R_{r}\left(x_{0},\Delta_{0}\right)}({\hat{\Psi }}_{i}-\Psi \left(z_{i};x_{i}\right){)}^{2}\;\;\;\;r=1,2,3,$$

and the marginal MSE approximations are computed as follows:

$$\hat{\text{MSE}}({\hat{\Psi }}_{i})=\frac{1}{1000}\sum_{i=1}^{1000}({\hat{\Psi }}_{i}-\Psi \left(z_{i};x_{i}\right){)}^{2},$$

where ${\hat{\Psi }}_{i}={\hat{\Psi }}_{i}\left(z\right)$ for the CRC method and ${\hat{\Psi }}_{i}={\hat{\Psi }}_{i}\left(z_{i}^{{\hat{\Delta }}_{0i}^{⋆}}\right)$ for the ARC method. The relative MSE of the two estimators is a convenient measure for comparing the MSEs. The conditional and marginal relative MSEs are denoted by

$${\text{ReMSE}}_{\text{cond}}=\frac{\hat{\text{MSE}}({\hat{\Psi }}_{i}\left(z_{i}^{{\hat{\Delta }}_{0i}^{⋆}}\right)|R_{r}\left(x_{0},\Delta_{0}\right))}{\hat{\text{MSE}}({\hat{\Psi }}_{i}\left(z\right)|R_{r}\left(x_{0},\Delta_{0}\right))}\;\;\text{and}\;\;{\text{ReMSE}}_{\text{marg}}=\frac{\hat{\text{MSE}}({\hat{\Psi }}_{i}\left(z_{i}^{{\hat{\Delta }}_{0i}^{⋆}}\right))}{\hat{\text{MSE}}({\hat{\Psi }}_{i}\left(z\right))},$$ (18)

respectively. From Fig 2, we observe that the performance of the ARC method depends on the Δ_0_ values and the region of the covariates. When ${\bar{\pi }}_{0}\in \left[0.60,0.95\right]$, increasing the value of Δ_0_ results in a smaller MSE approximation for the ARC method in the regions $R_{1}\left(x_{0},\Delta_{0}\right)$ and $R_{3}\left(x_{0},\Delta_{0}\right)$ that follow the results in Theorem 1. Then, increasing ${\bar{\pi }}_{0}$, Fig 2(D) shows that the MSE approximation for the proposed ARC method is greater than that of the CRC method in the region $R_{2}\left(x_{0},\Delta_{0}\right)$, for some Δ_0_.

Fig 2 shows that the ARC method has a smaller marginal MSE approximation than that of the CRC method. From Table 1, if we consider the true prior probabilities for all SNPs to be independent of the covariate, that is, *π*_0_(*x*_*i*_) = *π*_0_ for *i* = 1,…, *N*, then the CRC method has a smaller MSE than that of the ARC method. In such cases, the CRC method should be used instead of the ARC method to analyze the data.

**Table 1:** MSE of the ARC method relative to the CRC method when there is no covariate effect. The true prior probabilities are constant, *π*_0_(*x*_*i*_) = *π*_0_. The log_2_ value is given for the marginal relative MSE. Under the ARC method, Δ_0_ is 0.01.

*π*_0_	0.60	0.80	0.90	0.95
ReMSE_marg_	0.9030	0.8156	1.3729	2.0908

### 3.2 Real data analysis

We apply both the ARC and CRC methods to the CAD and DTI datasets, and compare the disease-associated SNPs and dyslexic-non-dyslexic difference voxels under each method, respectively. The purpose of this comparison is to demonstrate the practical difference between the methods rather than to determine which method performs better.

#### 3.2.1 CAD data analysis

The CAD dataset originating from the United Kingdom includes 500, 568 SNPs genotyped for 2, 000 cases, and 3, 000 combined on 22 autosomal chromosomes. The control individuals come from two groups: 1500 individuals from the 1958 British Birth Cohort (58C), and 1500 individuals selected from UK blood services (UKBS) controls. Following [12], we use quality control filtering methods to exclude SNPs based on the exact Hardy-Weinberg equilibrium (HWE) test as well as individuals or SNPs with many missing genotypes. The following three filters are applied sequentially. For the first filter, an SNP fails when the proportion of missing data proportion is greater than 0.05 or when the minor allele frequency (MAF) is smaller than 0.05 and the missing data proportion is greater than 0.01. For the second filter, SNPs with *p*-values smaller than 0.05 using the exact HWE test in the combined controls are rejected. Finally, for the third filter, we reject SNPs with *p*-values smaller than 5 × 10^−7^ using trend tests and general genotype tests between each case and the combined controls. We also excluded SNPs with MAFs smaller than 0.01. A total of *N* = 394839 SNPs passed these quality control filtering methods and are used to identify disease-associated SNPs. We apply both the ARC and the CRC methods to the CAD data and compare the disease-associated SNPs under each method.

**Fig 3:**
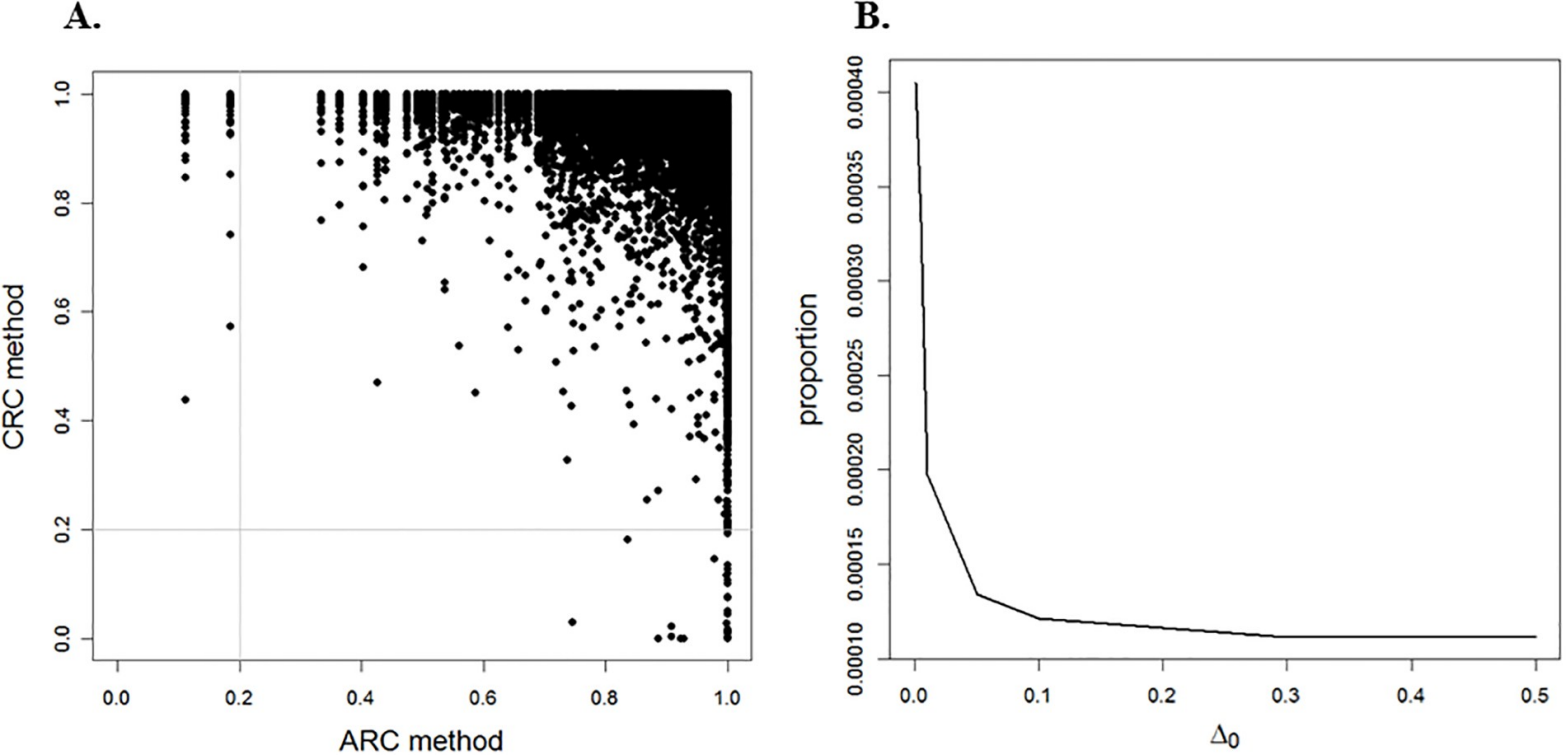
CAD data analysis. (A) presents the LFDR estimate under the ARC method for Δ_0_ = 0.001 versus that for the CRC method and (B) illustrates the proportion of disease-associated SNPs under the ARC method when the LFDR estimate is less than 0.2 versus Δ_0_ ∈ (0, 0.50).

The CAD related data introduced in Section 1.1, with *N* = 394, 839 SNPs, is employed in the following statistical analysis. Under the CRC method, all observed statistics ***z*** are considered to estimate the LFDR, where 44 disease-associated SNPs are identified with LFDR lower than 0.2. The MAF is incorporated as a covariate. Under the ARC method, the optimal reference class is estimated for each MAF, which depends on the choice of Δ_0_. Fig 3(A) presents the LFDR estimates under the CRC method versus the ARC method when Δ_0_ = 0.001. The results show that, 160 SNPs are disease-associated based on the ARC method, while the CRC method detects 44 disease-associated SNPs. From Fig 3(B), we find that changing Δ_0_ has a direct effect on the number of disease-associated SNPs. Under the ARC method, increasing the value of Δ_0_ brings the proportion of disease-associated SNPs closer to the corresponding proportion under the CRC method.

#### 3.2.2 DTI data analysis

Schwartzman et al. [5] used advanced MRI technology, DTI, to measure water diffusion in the human brain by scanning the brain. DTI is used to map and characterize the three-dimensional diffusion of a water molecule randomly moving in brain tissue to provide information regarding the direction of diffusion. The measured diffusivity; that is, the diffusion coefficient, relates the diffusive flux to a concentration gradient [27], and has units of (mm^2^/s). In this study, 12 children were tested and divided equally in each group (i.e., dyslexic or non-dyslexic group). Each child received DTI brain scans in N = 15443 locations, with each represented by its own voxel’s response. The aim is to determine the dyslexic-non-dyslexic difference at the *i*^th^ voxel (location), in relation to reading development in children aged 7-13 [28]. Each test corresponds to a specific voxel. We have two components (*z*_*i*_, *x*_*i*_) associated with each hypothesis for i = 1,…,N, where *z*_*i*_ is an observed test statistic that compares the dyslexic children with those who are not (see in Section 2), and *x*_*i*_ is the location (i.e., the distance from back of brain to the front). We apply both the ARC and CRC methods to the DTI data and compare the dyslexic-non-dyslexic difference voxels under each method. The DTI brain scans data with a total of N = 15443 locations, each represented by its own voxel’s response, is employed in the following statistical analysis.

**Fig 4:**
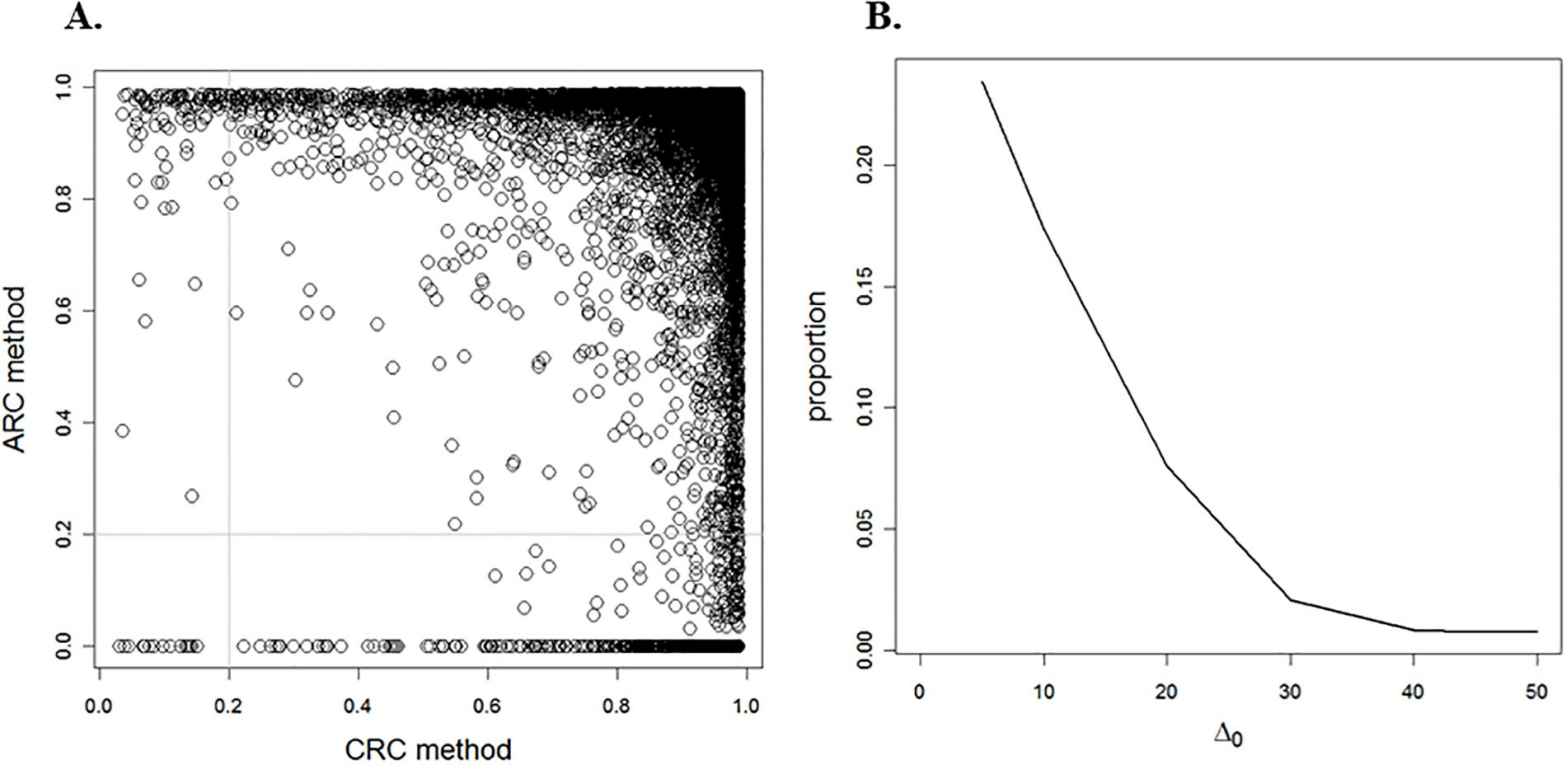
Brain data analysis. (A) presents the LFDR estimate under the ARC method for Δ_0_ = 20 versus that for the CRC method and (B) illustrates the proportion of dislexic-non-dyslexic difference voxels under the ARC method when the LFDR estimate is less than 0.2 versus Δ_0_ ∈ (0, 50).

Let $W_{i}={Z_{i}}^{2}$. The observed statistics ***w*** = (*w*_1_, *w*_2_,…, *w*_*N*_)^*T*^ are considered as a realization of ***W*** = (*W*_1_, *W*_2_,…, *W*_*N*_)^*T*^. Under the *i*^th^ null hypothesis, it holds that *W*_*i*_ ∼ *χ*_1_, while under the *i*^th^ alternative hypothesis *W*_*i*_ ∼ *χ*_1,*δ*_, where *δ* ∈ (0, inf) is an unknown noncentrality parameter, according to the models employed in [23, 24]. Therefore, the LFDR in Eq (1) is defined as follows

$$\Psi (w_{i})=\text{P}(A_{i}=0|W_{i}=w_{i})=\frac{\pi_{0}g_{0}\left(w_{i}\right)}{g(w_{i};\pi_{0},\delta )},$$ (19)

where *g*_0_(*w*_*i*_) ∼ *χ*_1_ (i.e., null density), and *g*(*w*_*i*_; *π*_0_, *δ*) denotes the mixture density given by

$$g(w_{i};\pi_{0},\delta )=\pi_{0}g_{0}\left(w_{i}\right)+\left(1-\pi_{0}\right)g_{1}\left(w_{i};\delta \right),$$ (20)

where *g*_1_(*w*_*i*_; *δ*) represents the unknown alternative density. Under the CRC method, all observed statistics ***w*** are considered to estimate the LFDR using the *type II maximum likelihood estimation* (MLE) model [24]

$$l\left(\pi_{0},\delta \right)=\sum_{i=1}^{N}\text{log}(\pi_{0}g_{0}\left(w_{i}\right)+\left(1-\pi_{0}\right)g_{1}\left(w_{i};\delta \right)),$$ (21)

where ${\hat{\pi }}_{0}=0.923$ and $\hat{\delta }=3.7$, and 119 dyslexic-non-dyslexic difference voxels are identified with LFDR lower than 0.2. Then, the brain location is incorporated as a covariate. Under the ARC method, the optimal reference class is estimated for each location, which depends on a choice of Δ_0_. Fig 4(A) presents the LFDR estimates under the CRC method versus the ARC method when Δ_0_ = 20. We observe from Fig 4(B), that changing Δ_0_ has a direct effect on the number of dyslexic-non-dyslexic difference voxels. Under the ARC method, increasing the value of Δ_0_ brings the proportion of dyslexic-non-dyslexic difference voxels closer to the corresponding proportion under the CRC method.

## 4 Discussion and conclusion

In this study, we employ a novel approach that incorporates a covariate (i.e., a scientific context corresponding to each hypothesis test) to improve the LFDR estimate when identifying alternative hypotheses. Using this approach, both the test statistic distribution under the alternative hypothesis and the prior probability that the null hypothesis is true, are modulated by the covariate. In the case where the prior probability *π*_0_(*X*_*i*_) is the step function given in Eq (16), Theorem 1 states that the ARC method has an MSE asymptotically no greater than that of the CRC method. It would be interesting to investigate whether this result holds for a general prior probability. We leave this topic for future research. In addition, the simulation indicates that the ARC method performs in comparison to the CRC method for a finite number of hypotheses. Our simulation results confirm that for regions $R_{1}\left(x_{0},\Delta_{0}\right)$ and $R_{3}\left(x_{0},\Delta_{0}\right)$, the LFDR estimator associated with the ARC method has a smaller MSE approximation than that of the CRC method (see Fig 2). Moreover, we could not prove the weak consistency of $\hat{\mu }\left(\Delta_{0},B\right)$ as an estimator of *π*_0_(*x*_*i*_) in region $R_{2}\left(x_{0},\Delta_{0}\right)$ (see S1 File, Lemma 2). The ARC method was applied to both CAD and DTI datasets, as illustrated in Figs 3 and 4. Regardless of LFDR estimation methods (i.e., HB and MLE), by increasing the value of the tuning parameter Δ_0_, the proportion of significant null hypotheses decreases, and approaches the proportion based on the CRC method. This suggests that further investigation may be necessary on how the tuning parameter Δ_0_ can be controlled to improve results.

## Supporting information

S1 File The proof of Theorem 1 proceeds by a series of lemmas. (PDF)
