# Supplementary material for: Estimating the local false discovery rate via a bootstrap solution to the reference class problem
Source: PLoS One. 2018 Nov 26;13(11):e0206902. doi: 10.1371/journal.pone.0206902 (PMC6261018; doi:10.1371/journal.pone.0206902)
Supplement: S1 File — (PDF) [file pone.0206902.s001.pdf]

Appendix

The proof of Theorem proceeds by a series of lemmas.

Lemma 1 *If the random indicator A_i that is conditional on $X_i = x_i$ is Bernoulli$\big(1-\pi_0(x_i)\big)$, then $E\big(\Psi(z_i;x_i)|X_i = x_i\big) = \pi_0(x_i)$.*

Proof 1 *We have*

$$E(A_i|Z_i = z_i, X_i = x_i) = P(A_i = 1|Z_i = z_i, X_i = x_i) = 1 - \Psi(z_i;x_i).$$

By taking the expectation with respect to the density of Z_i that is conditional on $X_i = x_i$, we obtain

$$\begin{aligned} E\big(E(A_i|Z_i = z_i, X_i = x_i)|X_i = x_i\big) =& E\big(1 - \Psi(z_i;x_i)|X_i = x_i\big), \\ E(A_i|X_i = x_i) =& 1 - E\big(\Psi(z_i;x_i)|X_i = x_i\big), \end{aligned}$$

and the result follows.

Lemma 2 *For $x_i \in \mathcal{R}_1(x_0, \Delta_0)$, the bootstrap estimator $\widehat{\mu}(\Delta_0, B)$ is a weakly consistent estimator of π_{01}. That is,*

$$\lim_{N\to\infty}\lim_{B\to\infty} P\big(|\widehat{\mu}(\Delta_0, B) - \pi_{01}| > \epsilon|X_i = x_i\big) = 0 \ \textit{ for any } \epsilon > 0.$$

Proof 2 *By Markov's inequality, it holds for any $\epsilon > 0$ that*

$$\begin{aligned} P\big(|\widehat{\mu}(\Delta_0, B) - \pi_{01}| > \epsilon|X_i = x_i\big) \leq& \frac{E\big[|\widehat{\mu}(\Delta_0, B) - \pi_{01}|\big|X_i = x_i\big]}{\epsilon} \\ \leq& \frac{E\big[|\widehat{\mu}(\Delta_0, B) - \mu_{\Delta_0}(x_i)|\big|X_i = x_i\big]}{\epsilon} \\ +& \frac{E\big[|\mu_{\Delta_0}(x_i) - \pi_{01}|\big|X_i = x_i\big]}{\epsilon}. \end{aligned}$$

$\widehat{\mu}(\Delta_0, B)$ is an unbiased estimator of $\mu_{\Delta_0}(x_i)$, and has zero variance as B becomes large. That is,

$$\lim_{B\to\infty} E\big[\big(\widehat{\mu}(\Delta_0, B) - \mu_{\Delta_0}(x_i)\big)^2|X_i = x_i\big] = \lim_{B\to\infty}\frac{\sigma^2_{\Delta_0}(x_i)}{B} = 0. \tag{1}$$

Hence,

$$\lim_{B\to\infty} E\big[|\widehat{\mu}(\Delta_0, B) - \mu_{\Delta_0}(x_i)|\big|X_i = x_i\big] \leq \lim_{B\to\infty} E\big[\big(\widehat{\mu}(\Delta_0, B) - \mu_{\Delta_0}(x_i)\big)^2|X_i = x_i\big]^{\frac{1}{2}} = 0.$$

On the other hand, when $x_i \in \mathcal{R}_1(x_0, \Delta_0)$ the expected dimension of the reference class $\boldsymbol{z}_i^{\Delta_0}$ as N becomes large is $\lim_{N\to\infty} d_i^{\Delta_0} = \infty$. By applying the consistency assumption of $\widehat{\Psi}_i$ on the reference class $\boldsymbol{z}_i^{\Delta_0}$, we have that

$$\lim_{N\to\infty} P\big(|\widehat{\Psi}_i(\boldsymbol{z}_i^{\Delta_0}) - \Psi(z_i;x_i)| > \epsilon|X_i = x_i\big) = 0.$$

Because $|\widehat{\Psi}_i(\boldsymbol{z}_i^{\Delta_0}) - \Psi(z_i;x_i)| \leq 1$, the dominated convergence Theorem implies that

$$\lim_{N\to\infty} E\big[\widehat{\Psi}_i(\boldsymbol{z}_i^{\Delta_0}) - \Psi(z_i;x_i)|X_i = x_i\big] = 0.$$

For $x_i \in \mathcal{R}_1(x_0, \Delta_0)$*,* $E\big(\Psi(z_i; x_i)|X_i = x_i\big) = \pi_{01}$ *and*

$$\lim_{N\to\infty} \frac{E\big[|\mu_{\Delta_0}(x_i) - \pi_{01}|\big|X_i = x_i\big]}{\epsilon} = 0.$$

Lemma 3 *If* $x_i \in \mathcal{R}_1(x_0, \Delta_0)$*, then the bootstrap estimator* $\widehat{\mathcal{B}}(\Delta, \Delta_0, B)$ *is a weakly consistent estimator of the prediction bias* $\mathcal{B}_\Delta(x_i)$*. That is,*

$$\lim_{N\to\infty} \lim_{B\to\infty} P\big(|\widehat{\mathcal{B}}(\Delta, \Delta_0, B) - \mathcal{B}_\Delta(x_i)| > \epsilon|X_i = x_i\big) = 0 \text{ for any } \epsilon > 0.$$

Proof 3 *By Markov's inequality, we have for any* $\epsilon > 0$ *that*

$$\begin{aligned} P\big(|\widehat{\mathcal{B}}(\Delta, \Delta_0, B) - \mathcal{B}_\Delta(x_i)| > \epsilon|X_i = x_i\big) &\leq \frac{E\big[|\widehat{\mathcal{B}}(\Delta, \Delta_0, B) - \mathcal{B}_\Delta(x_i)|\big|X_i = x_i\big]}{\epsilon} \\ &\leq \frac{E\big[|\widehat{\mu}(\Delta, B) - \mu_\Delta(x_i)|\big|X_i = x_i\big]}{\epsilon} \\ &+ \frac{E\big[|\widehat{\mu}(\Delta_0, B) - \pi_{01}|\big|X_i = x_i\big]}{\epsilon}. \end{aligned}$$

Because $\widehat{\mu}(\Delta, B)$ *is an unbiased estimator of* $\mu_\Delta(x_i)$ *whose variance is asymptotically zero, the result follows from Lemma 2 and the fact that*

$$\lim_{B\to\infty} E\big[|\widehat{\mu}(\Delta, B) - \mu_\Delta(x_i)|\big|X_i = x_i\big] \leq \lim_{B\to\infty} E\big[\big(\widehat{\mu}(\Delta, B) - \mu_\Delta(x_i)\big)^2|X_i = x_i\big]^{\frac{1}{2}} = 0.$$

Lemma 4 *For* $x_i \in \mathcal{R}_1(x_0, \Delta_0)$*, the bootstrap estimator* $\widehat{\Delta}^\star_{0i}$ *is a weakly consistent estimator of* $\Delta^\star_{0i}$*. That is,*

$$\lim_{N\to\infty} \lim_{B\to\infty} P\big(|\widehat{\Delta}^\star_{0i} - \Delta^\star_{0i}| > \epsilon|X_i = x_i\big) = 0 \text{ for any } \epsilon > 0.$$

Proof 4 *The bootstrap sample variance is a weakly consistent estimator of the variance of* $\widehat{\Psi}_i(\boldsymbol{z}_i^\Delta)$ *and it follows from Lemma 3, that*

$$\lim_{N\to\infty} \lim_{B\to\infty} P\big(|\widehat{err}\big(\Delta, \Delta_0, B\big) - err\big(\widehat{\Psi}(\boldsymbol{z}_i^\Delta)|X_i = x_i\big)| > \epsilon|X_i = x_i\big) = 0.$$

Therefore, the result follows from the continuous mapping Theorem and the fact that

$$\lim_{N\to\infty} \lim_{B\to\infty} P\big(|\arg\inf_{\Delta\geq\Delta_0} \widehat{err}\big(\Delta, \Delta_0, B\big) - \arg\inf_{\Delta\geq\Delta_0} err\big(\widehat{\Psi}(\boldsymbol{z}_i^\Delta)|X_i = x_i\big)| > \epsilon|X_i = x_i\big) = 0.$$

Proof of Theorem:

Proof 5 *We know that*

$$MSE\big(\widehat{\Psi}_i(\boldsymbol{z}_i^{\widehat{\Delta}^\star_{0i}})|\mathcal{R}_1(x_0,\Delta_0)\big) = \int_{\mathcal{R}_1(x_0,\Delta_0)} MSE\big(\widehat{\Psi}_i(\boldsymbol{z}_i^{\widehat{\Delta}^\star_{0i}})|X_i = x_i\big)\, dP_{x_i},$$

$$MSE\big(\widehat{\Psi}_i(\boldsymbol{z})|\mathcal{R}_1(x_0,\Delta_0)\big) = \int_{\mathcal{R}_1(x_0,\Delta_0)} MSE\big(\widehat{\Psi}_i(\boldsymbol{z})|X_i = x_i\big)\, dP_{x_i}.$$

It suffices to show that

$$\lim_{N\to\infty}\lim_{B\to\infty}\Big[MSE\big(\widehat{\Psi}_i(\boldsymbol{z}_i^{\widehat{\Delta}^\star_{0i}})|X_i = x_i\big) - MSE\big(\widehat{\Psi}_i(\boldsymbol{z})|X_i = x_i\big)\Big] \le 0.$$

$$\begin{aligned} MSE\big(\widehat{\Psi}_i(\boldsymbol{z}_i^{\widehat{\Delta}^\star_{0i}})|X_i = x_i\big) - MSE\big(\widehat{\Psi}_i(\boldsymbol{z})|X_i = x_i\big) =& err\big(\widehat{\Psi}_i(\boldsymbol{z}_i^{\widehat{\Delta}^\star_{0i}})|X_i = x_i\big) \\ & - err\big(\widehat{\Psi}_i(\boldsymbol{z}_i^{\Delta^\star_{0i}})|X_i = x_i\big) \\ & + err\big(\widehat{\Psi}_i(\boldsymbol{z}_i^{\Delta^\star_{0i}})|X_i = x_i\big) \\ & - err\big(\widehat{\Psi}_i(\boldsymbol{z})|X_i = x_i\big). \end{aligned}$$

From Lemma 4, the weak consistency of $\widehat{\Delta}^\star_{0i}$ implies that

$$\lim_{N\to\infty}\lim_{B\to\infty} err\big(\widehat{\Psi}_i(\boldsymbol{z}_i^{\widehat{\Delta}^\star_{0i}})|X_i = x_i\big) - err\big(\widehat{\Psi}_i(\boldsymbol{z}_i^{\Delta^\star_{0i}})|X_i = x_i\big) = 0.$$

On the other hand, because $\Delta^\star_{0i}$ is optimal tuning parameter, it follows that

$$err\big(\widehat{\Psi}_i(\boldsymbol{z}_i^{\Delta^\star_{0i}})|X_i = x_i\big) - err\big(\widehat{\Psi}_i(\boldsymbol{z})|X_i = x_i\big) \le err\big(\widehat{\Psi}_i(\boldsymbol{z}_i^{\Delta})|X_i = x_i\big) - err\big(\widehat{\Psi}_i(\boldsymbol{z})|X_i = x_i\big)$$

for any $\Delta \in [\Delta_0, \infty)$, which indicates that

$$\begin{aligned} &\lim_{N\to\infty}\lim_{B\to\infty}\big[MSE\big(\widehat{\Psi}_i(\boldsymbol{z}_i^{\widehat{\Delta}^\star_{0i}})|X_i = x_i\big) - MSE\big(\widehat{\Psi}_i(\boldsymbol{z})|X_i = x_i\big)\big] \\ &\qquad \le \lim_{N\to\infty}\lim_{B\to\infty}\big[err\big(\widehat{\Psi}_i(\boldsymbol{z}_i^{\Delta})|X_i = x_i\big) - err\big(\widehat{\Psi}_i(\boldsymbol{z})|X_i = x_i\big)\big] \\ &\qquad = \lim_{N\to\infty}\lim_{B\to\infty}\big[MSE\big(\widehat{\Psi}_i(\boldsymbol{z}_i^{\Delta})|X_i = x_i\big) - MSE\big(\widehat{\Psi}_i(\boldsymbol{z})|X_i = x_i\big)\big] = 0. \end{aligned}$$

The facts that both $\lim_{N\to\infty} MSE\big(\widehat{\Psi}_i(\boldsymbol{z})|X_i = x_i\big) = 0$ and $\lim_{N\to\infty} MSE\big(\widehat{\Psi}_i(\boldsymbol{z}_i^{\Delta})|X_i = x_i\big) = 0$ follow from the consistency and the dominated convergence Theorem.
